# Stressed by Maternity: Changes of Cortisol Level in Lactating Domestic Cats

**DOI:** 10.3390/ani10050903

**Published:** 2020-05-22

**Authors:** Galina S. Alekseeva, Julia A. Loshchagina, Mariya N. Erofeeva, Sergey V. Naidenko

**Affiliations:** 1 A.N. Severtsov Institute of Ecology and Evolution, Russian Academy of Sciences, 119071 Moscow, Russia; erofeevamariya@yandex.ru (M.N.E.); snaidenko@mail.ru (S.V.N.); 2 Institute of Geography, Russian Academy of Sciences, 119017 Moscow, Russia; julia.loshchagina@gmail.com

**Keywords:** glucocorticoids, cortisol, lactation, reproductive success, litter size, cat

## Abstract

**Simple Summary:**

Milk feeding is the most important part of maternal care in the first weeks of the offspring’s life as it contributes to growth and development of the young, but at considerable energetic expense to the mother. One of the indicators that can be used to assess the physiological state of the female is the cortisol level relating to the stress of animals. Presumably, the more offspring there are in the litter, the higher the level of stress on the mother and, accordingly, the higher the cortisol concentrations. This study presents our data obtained in domestic cats whose litter size varied from 1 to 7 kittens. We found that the highest cortisol concentrations were observed at the peak of lactation, i.e., in 4 weeks of kittens’ life, when the offspring’s milk needs were at maximum. Moreover, during the period of offspring’s growth, the hormone level was higher in females with 1–3 kittens. In addition, cortisol concentrations in less productive cats were very high, even before mating.

**Abstract:**

Lactation is the most energetically expensive component of maternal care in mammals. Increased reproductive investment can lead to physiological stress for the mothers, based on the exhaustion of energy resources and increase in glucocorticoids level. This study aimed to estimate the changes in cortisol concentrations during lactation in domestic cats and compared the differences among litter sizes. Eleven females gave birth to 27 litters, which were divided in two groups—small (1–3 kittens) and large (4–7 kittens) litters. Blood samples were collected from each female before mating, after parturition, at 4 and 8 weeks of lactation. We showed that the cortisol level in females changed significantly during lactation—the highest concentrations were observed at the peak of lactation at 4 weeks. Cortisol levels varied significantly among females but did not depend on their maternal experience. We also revealed that there were no differences in cortisol levels between females with small and large litters, but at 4 weeks of lactation, the hormone concentrations were higher in females with small litters. It is likely that these females initially invested less in reproduction, giving birth to fewer offspring.

## 1. Introduction

In mammals, pregnancy and lactation are the most energetically costly processes that are of paramount importance to the survival, development, and growth of their young [1,2]. Daily energy expenditures of lactating females increase up to 150% over non-reproductive females, and are directly related to litter size [1,3]. Females of most species accumulate energy in body stores during pregnancy and deplete body stores during lactation [4,5].

The exhaustion of energy resources associated with maternal care can lead to physiological stress in females [6]. In vertebrates, the most used parameter of stress assessment is the level of glucocorticoids (cortisol or corticosterone) in blood serum and different excretions [7,8,9,10,11]. Changes in glucocorticoid concentrations were also noted in connection to increasing load on the organism, due to physiological stress and requirement to mobilize internal resources [12,13]. Furthermore, glucocorticoid level is a good indicator of metabolism intensity [7,14,15,16,17]. Experiments on different animal species have confirmed that an increase in glucocorticoid levels might be a consequence of parental efforts [18,19,20,21].

Variation in glucocorticoid concentrations can be partly imputed to differences in energetic requirements of animals [18]. In addition to reproduction itself, the reproductive cost is dependent on litter size, as females with large litters experience heavier loads during pregnancy and lactation [3,21,22,23,24]. Females of some rodents are able to regulate the litter size according to their own physiological status and available resources [23,25]. Increased reproductive investment, i.e., the production of more numerous or heavier offspring, might elicit higher glucocorticoid levels in females [18,21,26].

There are only a few studies considering changes in glucocorticoid levels during the young rearing period. The number of cubs per litter is rarely described. Most of the available data are from bird species [18,27,28] or selected groups of mammals (mainly rodents and primates) [26,29,30,31]. In this study, we aimed to explore changes in cortisol levels in female cats, during lactation, and relate it to litter size. The domestic cat (*Felis catus*) might be a useful model for research of maternal physiological adjustments (including endocrinological mechanisms) of parturition and lactation, both in wild cats and in other mammals. Domestic cats can reproduce up to three times per year with litter sizes from 1 to 8 kittens [22,32,33]. Kittens are completely dependent on maternal milk during the first weeks of life [33,34]. At the age of one month, the kittens start eating solid food [34]. Nursing of kittens stops approximately in the eleventh week of their life [35]. Maternal energy intake in domestic cat increases 1.5–1.7 times during pregnancy and 2.5–3.0 times during kitten rearing periods, especially at the peak of lactation at 4–5 weeks [36]. In addition, the litter size affects the growth rate of kittens. The more kittens there are in a litter, the less their weights are at birth and during the first weeks of life [22,37].

We tested two related hypotheses. The first was that the cortisol level in female cats should be the highest at the period of maximum energy requirement for females [36], i.e., before the kittens change from milk to solid food. Second, female cats with large litters should have higher cortisol levels during lactation than the ones with small litters because of the higher energetic expenditure required for raising offspring [22].

## 2. Materials and Methods

### 2.1. Husbandry Conditions and Animals

The study was conducted in 2011–2015 and 2018 at the Joint Usage Center “Live collection of wild species of mammals” of the A.N. Severtsov Institute of Ecology and Evolution (Tchernogolovka, Russia) situated 50 km north-east of Moscow, Russia (56°00′ N, 38°22′ E). All domestic cats were outbred cats, which allowed us to avoid artificial genetical restrictions. We knew all founders of our experimental population, managed pedigree of cats and prevented inbred mating. Females were mated only once a year in March–April (to eliminate effects of previous litter and season). We added the male-to-female during estrus period that was determined by the typical behavior patterns in cats [38]. All females gave birth in May–June, so we finished blood collecting at the end of summer, which conformed to the period of optimal environmental conditions for breeding cats [39,40].

All females were housed outdoors together with their litters, separate from other conspecifics, being exposed to natural temperature fluctuations and photoperiod, during the research. Average air temperature in May–August was 15–19 °C (maximum 21–25 °C and minimum 8–12 °C). The animals were kept in wire-meshed enclosures with a partly natural environment and a wooden box, as a shelter in each enclosure. The size of the enclosure was 4 m^3^ (volume = 2 m (length) × 1 m (width) × 2 m (height)). The cats were fed daily (six times a week, and fasted for one day). The daily ration consisted of about 400 g of chicken parts or mincemeat with additional vitamins, according to the food demand of individuals. The diet of females with suckling kittens was about 1.5 times higher, depending on the litter size. The animals had water access ad libitum. Since the domestic cat is a model species for studying the reproductive biology of the wildcat species in the Joint Usage Center, the animals of all species were kept under the same conditions [11,37].

Altogether, we used 11 females that gave birth to 27 litters with a total number of 101 kittens (Table 1). Only 5 out of 27 litters were obtained from young primiparous females (1–2 years). In all other cases, females were 3–6 years old and had 2–6 litters in their life. The average body mass of females before mating, was 3.16 ± 0.06 kg and did not differ during lactation, depending on the number of kittens in litters [41]. Litter size varied from 1 to 7 after parturition (3.74 ± 0.28). The kitten mortality rate was 27%, of which 16% were stillborn and 11% died during lactation. There were no significant differences in the mortality rate in small and large litters. There were not enough litters of different size to consider the litter size as a continuous variable. Deag et al. [22] found body mass differences in domestic kittens from litters that were larger and smaller than the mean litter size. As a result, we also divided all litters in two groups, according to the mean litter size for our dataset—small litters (11 with 1–3 kittens) and large litters (16 with 4–7 kittens).

### 2.2. Blood Sampling

The blood sampling procedure was performed using manual restraint. Samples were collected from each female at four points—(1) immediately before mating (same day as exposure to male), (2) after parturition (within 1–2 days; 0 weeks of lactation), (3) at 4 weeks of lactation, and (4) at 8 weeks of lactation. The blood (0.5–1 mL) was taken from the femoral vein into Eppendorf tubes (SSI, Lodi, CA, USA) in the morning (9:00–12:00 a.m.), to minimize the effect of daily fluctuations in hormone concentrations. The time interval between animal capture and blood sampling was no more than 3 min, which allowed us to consider the cortisol level in the blood serum as basal [42,43]. Blood samples were centrifuged (20 min at the rate 6000 rpm) immediately after sampling and the serum was transferred into new clear Eppendorf tubes, frozen, and stored at −18 °C, until hormone analysis.

### 2.3. Cortisol Assay

Laboratory analysis for cortisol was conducted by enzyme immunoassay (EIA). Serum samples were thawed, and cortisol concentrations were measured with commercial enzyme immunoassay kits (Immunotech, Moscow, Russia), following the manufacturer guidelines. Cortisol antibody cross-reactivity to other steroids was 6% to prednisolone and <1% for all other tested steroids. We used the flatbed spectrophotometer Multiscan EX with the Ascent Software v.2.6 (Thermo Fisher Scientific, Vantaa, Finland) for measuring the optical density in the wells at 450 and 620 nm and comparing it with the standard values. Serum samples were analyzed in duplicates (*n* = 220), and the coefficient of variation (CV) was calculated. If the CV was more than 10%, the samples were re-assayed. When CV was less than 10%, the mean cortisol concentration was taken for further analysis. The interassay coefficient of variation for the standard concentration of 7.25 ng/mL was 1.99% (*n* = 6) (n is the number of used plates). The mean intraassay coefficient of variation of paired samples was 3.03 ± 0.19% (*n* = 173) (*n* is the number of measured samples). All details are shown in Table S1.

### 2.4. Statistical Analysis

Statistical analysis was performed using the R software version 3.5.1 (R Core Team, Vienna, Austria) [44]. We used histograms and dotchart plots for visual analysis of the data structure and for identifying outliers [45]. To normalize distributions, cortisol concentrations were log10 transformed. To analyze the dynamics of cortisol level in females, during the lactation period, we applied linear mixed-effects models [46] implemented in the lme4 package [47]. We included the following fixed factors into the model—sample time (before mating, 0 weeks of lactation, 4 weeks of lactation, and 8 weeks of lactation), litter size (large or small), maternal experience (multiparous or primiparous). Female code and year were included into the model as random factors to deal with repeated litters from some females in different years [46]. Including random slopes for maternal experience and number of kittens per litter degraded our model—Akaike information criterion (AIC) of these models was higher (69.26 and 71.52) than AIC of the random intercept model (67.55), on the condition that the fixed parts of the models were the same.

To find the optimal fixed part of the model, we used the top-down strategy [46]. The significance of the fixed factors was estimated by excluding these factors from the final model, in turn, and comparing derived models with the final model. *p*-values were obtained by likelihood ratio tests of *anova* command of the full model with the effect in question against the model, and without the effect in question. The model fit was tested by visual estimation of residual plots [46], which had no signs of violation of the underlying assumptions. Owing to the significance of sample time, we used the *lsmeans* package [48] to perform post-hoc comparisons and find which points differed from each other.

To get more detailed information about differences between females with small and large litters at different sampling points, we applied an additional model that besides sample time and litter size, included their interaction. Female code and year were also included as random intercepts into this model. Pairwise comparisons of the cortisol concentrations between the females with small and large litters at different sample times and between sampling points in each litter size group were performed using the *lsmeans* function in the *lsmeans* package [48].

To perform analysis of differences in cortisol concentrations in females with a different number of kittens per litter, we included the actual number of kittens per litter (from 1 to 7) into the final model instead of the litter size (small or large). Pairwise comparisons of cortisol concentrations between females with a different number of kittens in the litter were conducted using the *lsmeans* function in the *lsmeans* package [48]. All data are presented as the mean values and standard errors of the mean (M ± SE).

### 2.5. Ethical Approval

All experimental procedures had no apparent adverse effects on the animals. After the collection of the blood samples, the individuals moved and behaved normally. No drop in body mass was recorded in subsequent weighings. No special permission for use of cats in behavioral research is required in the Russian Federation. The relevant ethic committee that regulates research on animals in the A.N. Severtsov Institute of Ecology and Evolution of the Russian Academy of Sciences is the Commission on Regulatory of Experimental Research (Bioethics Commission) of the A.N. Severtsov Institute of Ecology and Evolution of the Russian Academy of Sciences (IEE RAS). It was only created in 2017, so it was not possible to get the permission for the first part of the study. However, this committee provided permission for this project (permission no. 21 of 24/04/2018). The study was conducted in accordance with the ASAB/ABS (the Association for the Study of Animal Behaviour and the Animal Behavior Society) Guidelines for the Treatment of Animals in Behavioural Research and Teaching [49], and in accordance with the laws of Russian Federation, the country where the research was performed.

## 3. Results

Cortisol concentrations in domestic cats varied from 6 ng/mL to 995 ng/mL, considering sampling time and litter size. All values were within the interval between the lower quartile—3 IQR (interquartile range) and 3 IQR + upper quartile, which allowed us to include all data into further statistical analysis. The final model described cortisol variation in domestic cats during the lactation period, including sample time and litter size, as fixed factors, and female code and year as random intercepts. The individual female had a strong significant effect on cortisol concentration—full model included random intercept was better than the full model without this random factor (Likelihood ratio test: L = 59.229, df = 1, *p* < 0.0001). The factor maternal experience was statistically non-significant, which allowed us to exclude it from the final model (Likelihood ratio test: χ^2^ = 2.23, df = 1, *p* = 0.6294).

**Figure 1:**
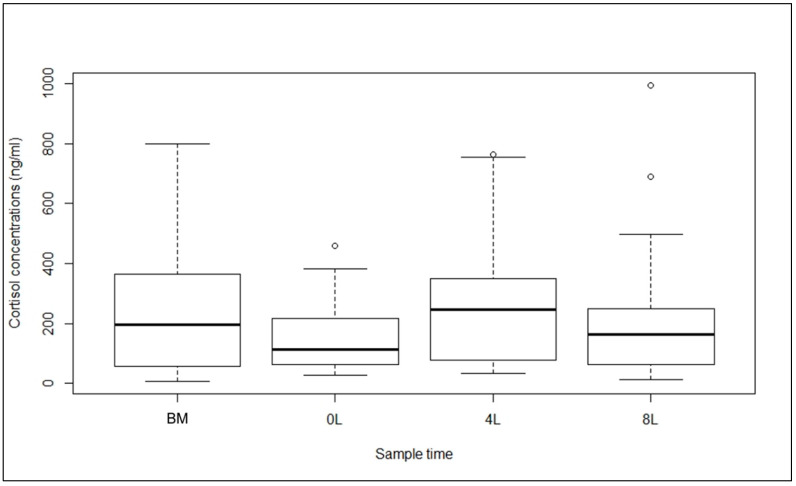
Cortisol concentrations in females of the domestic cat at different sampling points: BM—before mating, 0L—after parturition (0 weeks of lactation), 4L—4 weeks of lactation, and 8L—8 weeks of lactation. Plotted are the median (horizontal line in the box), lower and upper quartiles (horizontal box boundaries), and minimum and maximum values (whiskers); dots indicate outliers.

The cortisol level changed significantly during the lactation period, in domestic female cats (Likelihood ratio test: χ^2^ = 12.64, df = 3, *p* = 0.0055). Cortisol concentrations were significantly higher at 4 weeks of lactation than after parturition (β = 0.23 ± 0.07, t = 3.48, *p* = 0.0008), at 8 weeks of lactation (β = 0.15 ± 0.07, t = 2.22, *p* = 0.029), and before mating (β = 0.18 ± 0.07, t = – 2.43, *p* = 0.017; Figure 1; Table S2).

Cortisol levels did not differ between females with small (1–3 kittens) and large (4–7 kittens) litters (Likelihood ratio test: χ^2^ = 2.02, df = 1, *p* = 0.155). Pairwise comparisons of cortisol concentrations in females with large and small litters at each sampling point showed that at 4 weeks of lactation, cortisol levels had a tendency to be higher in females with small litters than in ones with large litters (β = 0.198 ± 0.109, t = 1.822, *p* = 0.0719; Table S3). The cortisol concentrations from the basal level (before mating) to 4 weeks of lactation increased 2.5-fold (250 ± 111%) in females with small litters and less than 2-fold (194 ± 40%) in females with large litters.

**Figure 2:**
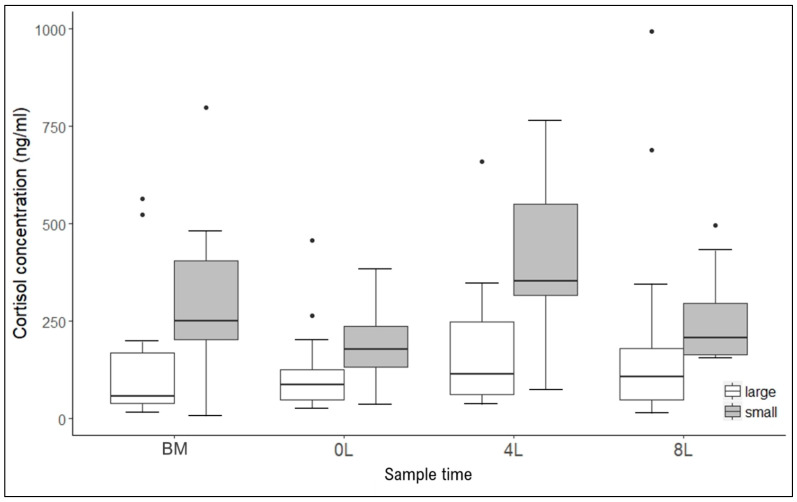
Cortisol concentrations in females of the domestic cat with small (grey boxes) and large (white boxes) litters at different sampling points. BM: before mating, 0L: after parturition (0 weeks of lactation), 4L: 4 weeks of lactation, and 8L: 8 weeks of lactation. Plotted are the median (horizontal line in the box), lower and upper quartiles (horizontal box boundaries), and minimum and maximum values, within the interval upper quartile +1.5 inter-quartile range and lower quartile −1.5 inter-quartile range (whiskers); black dots indicate outliers.

Cortisol dynamics during lactation was similar in females with different litter size (Figure 2; Table S4). In females with large litters, the cortisol dynamics was less pronounced—only a non-significant trend was observed between the point after parturition and 4 weeks of lactation (β = −0.16 ± 0.09, t = –1.84, *p* = 0.069). In females with small litters, cortisol concentrations were significantly higher at 4 weeks of lactation than after parturition (β = 0.336 ± 0.105, t = 3.195, *p* = 0.002), at 8 weeks of lactation (β = 0.227 ± 0.105, t = 2.166, *p* = 0.0333), and before mating (β = 0.222 ± 0.108, t = 2.054, *p* = 0.0432).

Pairwise comparisons of cortisol concentrations in females with a different number of kittens did not reveal a clear correlation of hormone level in females and their litter size (Figure S1). Only some differences were observed, in particular, cortisol concentrations in females with two kittens were significantly higher as compared to females with 5 kittens. Detailed results are shown in Tables S5 and S6.

## 4. Discussion

The approach of parturition in mammals is accompanied by an increase in the level of stress hormones, even if the female did not exhibit behavioral anxiety [29,31,50]. The activity of the hypothalamic-pituitary-adrenal (HPA) axis rose during the prenatal period [30]. Glucocorticoid concentrations increased before parturition in blood serum in laboratory rats [51] and dogs [52]. After parturition, corticosteroids level decreased quite rapidly during the first hours or days of lactation [29,31,52,53].

In our study, the cortisol levels in blood serum of lactating domestic cats was also minimal after parturition (during the 1st–2nd days of lactation) and close to the before-mating values. However, after that, cortisol concentrations rose, reaching maximum values by 4 weeks of lactation. This period coincided with the changes in diet—the kittens started to take solid food at this age, but they still mainly depended on mothers’ milk. Thus, our first hypothesis predicting the highest cortisol level in females at the peak of lactation was supported by our results. Presumably, the energetic requirements for the milk production were extreme during this period of lactation [36], which necessitated mobilization of additional energy and, accordingly, caused an increase in glucocorticoid concentrations. A similar hormonal pattern at the time when the offspring started to switch to solid food diet was also described in yellow-pine chipmunk (*Tamias amoenus*) [20] and fur seal (*Arctocephalus tropicalis*) [19] females. According to the results from lactating fur seals, the increase in cortisol levels accompanied by a decrease in body condition index of females, could either contribute to the mobilization of protein reserves to ensure milk production when easily mobilized reserves were used or could act as a re-feeding signal that was triggered long before the females exhausted their body store [19].

It is believed that the reproductive success of females is significantly dependent on their age [54,55,56]. Earlier studies in Antarctic fur seals (*Arctocephalus gazella*) and reindeers (*Rangifer tarandus*) have shown that the primiparous females give birth to fewer cubs with less body weight [56,57]. However, we did not detect any differences among females of the domestic cat with different maternal experience, as was also described for the wildcat species [58]. The majority of females in our study were multiparous (81.5%), but their litter size varied regardless of their experience. Individual characteristics of females mattered a lot to the intensity of maternal care [22,24], since the mothers could be in different physical conditions, and accordingly, could take care of their litters in different ways.

Parental-investment theory suggests that maternal effort is partially determined by the reproductive value of current offspring [59]. The number of offspring and their survival depends on the level of parental investment. Limited resources constrain parents to decide between provisioning their offspring and their own survival [60]. The total birth weight of litter in domestic cat is about 12% of maternal weight before mating, which is much higher than in wildcats [2,37]. Maternal energy intake during lactation is enhanced 2.5–3.0 times, especially, at the peak in 4–5 weeks [36]. A decline in the females’ body condition during lactation in the domestic cat has been described earlier [22]. Symptoms of stress, i.e., problems with coordination of movements, inability to stand on their paws, and poor coat, were detected in 27% of lactating females. Such signs of weak health were found only in females with large litters [22]. It suggests a higher stress level (and glucocorticoids concentrations) in females with larger litters. Our second hypothesis was based on these data and we expected that the females with the larger litters would have a higher cortisol level than the females with the smaller number of offspring.

However, we revealed that the average cortisol level did not differ between females with small and large litters. It was slightly higher in cat females with smaller litters over the study period, especially at 4 weeks of lactation. In addition, the cortisol concentrations in these females (which gave birth to small litters) were higher even before mating. These data did not support our hypothesis. Probably, such females were more stressed or with higher metabolisms, supposedly causing higher glucocorticoids concentration [15], and they initially invested less in reproduction, giving birth to fewer offspring. Increasing energetic requirements during lactation had more pronounced negative consequences for them than for females with lower basal cortisol level. Similar results were also obtained in rhesus monkeys (*Macaca mulatta*)—females with fewer infants secreted milk with a high cortisol level [26]. It was likely that the young in small litters might have thermal restrictions during the early stages of development that was described in the European rabbit (*Oryctolagus cuniculus*) [61]. Consequently, mothers with small litters need to spend more time with their kittens to keep them warm, which is ultimately more costly for the females.

## 5. Conclusions

By documenting the relationships between cortisol concentrations during lactation and litter size in domestic cats, our study suggests that measuring hormone fluctuations can provide an understanding of how females react to physiological stress during their lactation and how it is related to their reproductive success. We showed that the cortisol level in domestic cats changed significantly during lactation. The highest concentrations were observed at the peak of lactation (4 weeks), i.e., during the maximum energetic requirements for the milk production in lactating females. Possibly, cat breeders might prevent the highest lactation stress if they wean kittens after the third week of their life. At the same time, the individual female had a strong significant effect on cortisol concentration, and maternal experience did not have any effect. Our results also revealed that cortisol levels in females with small litters were slightly higher than in ones with large litters and the changes in cortisol concentrations during lactation were pronounced only in females with small litters. It is probable that dividing the data into three groups (small, 1–2 kittens; medium, 3–4 kittens; and large, 5 and more kittens) could make the differences in cortisol levels in females more obvious, but our sample size was insufficient for this type of analysis. In addition, none of our animals were restricted in terms of food, unlike feral cats, whose food resources are limited, which could also reveal clearer differences in cortisol levels in females with small and large litters. Further studies with more animals might answer these questions.

## Figures and Tables

**Table 1:** Females used in the study, their maternal experience, and litter size in different years.

Year of Study	Female Code	Maternal Experience	Number of Litter	Number of Kittens per Litter	Litter Size
**2011**	MK	primiparous	1	3	small
**2012**	BN	multiparous	3	4	large
	GR	multiparous	2	4	large
	MR	multiparous	3	7	large
	MS	multiparous	2	5	large
	MK	multiparous	2	2	small
**2013**	BN	multiparous	4	6	large
	GM	multiparous	4	4	large
	GR	multiparous	3	3	small
	MR	multiparous	4	2	small
	MS	multiparous	3	1	small
	MK	multiparous	3	3	small
	OR	primiparous	1	5	large
	PZ	multiparous	3	4	large
**2014**	FK	primiparous	1	3	small
	GM	multiparous	5	4	large
	GR	multiparous	4	3	small
	OR	multiparous	2	1	small
	PK	primiparous	1	4	large
**2015**	GM	multiparous	6	3	small
	GR	multiparous	5	5	large
	MK	multiparous	4	5	large
	OR	multiparous	3	4	large
**2018**	FK	multiparous	3	5	large
	PZ	multiparous	6	5	large
	PK	multiparous	3	4	large
	VR	primiparous	1	2	small

## Supplementary Materials

The following are available online at https://www.mdpi.com/2076-2615/10/5/903/s1. Table S1: Cortisol concentrations, body mass, and other characteristics of the domestic female cats used in the study in different years; Table S2: Pairwise comparisons of cortisol concentrations at different sampling points (before mating, after parturition, 4 weeks of lactation and 8 weeks of lactation) in the domestic female cats; Table S3: Pairwise comparisons of cortisol concentrations between the domestic female cats with small and large litters at different sampling points (before mating, after parturition, 4 weeks of lactation, and 8 weeks of lactation); Table S4: Pairwise comparisons of cortisol concentrations at different sampling points (before mating, after parturition, 4 weeks of lactation, and 8 weeks of lactation) in the domestic female cats with small and large litters; Table S5: Pairwise comparisons of cortisol concentrations between the domestic female cats with different number of kittens per litter; Table S6: Pairwise comparisons of cortisol concentrations between the domestic female cats with different number of kittens per litter at 4 weeks of lactation; Figure S1: Cortisol concentrations in females of the domestic cat with different number of kittens per litter.
