# Supplementary material for: Stressed by Maternity: Changes of Cortisol Level in Lactating Domestic Cats
Source: Animals (Basel). 2020 May 22;10(5):903. doi: 10.3390/ani10050903 (PMC7278448; doi:10.3390/ani10050903)
Supplement: Supplementary file 1 [file animals-10-00903-s001.pdf]

Table S1: Cortisol concentrations, body mass and other characteristics of females used in the study in different years.

No	Year of study	Female name	Maternal experience	Number of litter	Kittens number per litter	Litter size	Body mass, kg	Cortisol concentration, ng/ml			
								Sample time			
								before mating	0 weeks of lactation (after parturition)	4 weeks of lactation	8 weeks of lactation
1	2011	MK	primiparous	1	3	small	2,520	214,00	304,00	304,90	431,20
2	2012	BN	multiparous	3	4	large	3,330	24,93	35,56	33,60	27,53
3	2012	GR	multiparous	2	4	large	3,205	55,39	50,15	260,50	172,10
4	2012	MR	multiparous	3	7	large	3,560	141,20	111,70	244,60	345,80
5	2012	MS	multiparous	2	5	large	3,400	13,16	26,16	47,15	12,94
6	2012	MK	multiparous	2	2	small	2,730	481,00	236,90	372,30	207,90
7	2013	BN	multiparous	4	6	large	3,505	34,86	44,21	77,67	57,78
8	2013	GM	multiparous	4	4	large	2,580	564,40	113,70	348,90	148,82
9	2013	GR	multiparous	3	3	small	3,275	365,00	177,30	369,40	251,10
10	2013	MR	multiparous	4	2	small	3,600	217,40	382,50	352,60	163,66
11	2013	MS	multiparous	3	1	small	3,490	5,98	35,49	73,50	53,47
12	2013	MK	multiparous	3	3	small	2,850	798,80	149,84	764,20	110,10
13	2013	OR	primiparous	1	5	large	3,060	196,50	263,60	239,00	96,28
14	2013	PZ	multiparous	3	4	large	3,260	44,74	112,90	132,10	55,66
15	2014	FK	primiparous	1	3	small	2,890	198,40	145,20	199,70	163,00
16	2014	GM	multiparous	5	4	large	3,025	523,20	458,60	660,40	689,00
17	2014	GR	multiparous	4	3	small	3,475	417,40	230,50	326,60	249,80
18	2014	OR	multiparous	2	1	small	3,120	130,20	236,80	328,40	205,40
19	2014	PK	primiparous	1	4	large	2,975	58,07	73,44	77,44	118,90
20	2015	GM	multiparous	6	3	small	3,040		80,89	754,38	341,73
21	2015	GR	multiparous	5	5	large	3,450		51,96	95,05	66,16
22	2015	MK	multiparous	4	5	large	2,910		200,15	317,76	995,18
23	2015	OR	multiparous	3	4	large	3,200		157,74	213,77	195,24

24	2018	FK	multiparous	3	5	large	3,225		98,03	54,87	174,15
25	2018	PZ	multiparous	6	5	large	3,350	109,60	76,71	39,95	25,00
26	2018	PK	multiparous	3	4	large	3,460		32,81	62,89	28,56
27	2018	VR	primiparous	1	2	small	2,815	282,00	119,72	729,00	497,25
							3,16 ± 0,06 [1]	232,20 ± 47,48 [1]	148,39 ± 21,37 [1]	277,06 ± 43,10 [1]	217,92 ± 42,84 [1]

[1] Data are presented as mean ± SE.

Table S2. Pairwise comparisons of cortisol concentrations at different sampling points (before mating, after parturition, 4 weeks of lactation and 8 weeks of lactation) in the domestic cat females.

Contrast	Estimate	SE	t-ratio	*p*-value
before mating – after parturition	0.056	0.073	0.761	0.4486
before mating – 4 weeks of lactation	– 0.176	0.073	– 2.427	***0.0173***
before mating – 8 weeks of lactation	– 0.029	0.073	– 0.395	0.6938
after parturition – 4 weeks of lactation	– 0.232	0.067	– 3.483	***0.0008***
after parturition – 8 weeks of lactation	– 0.084	0.067	– 1.263	0.2101
4 weeks of lactation – 8 weeks of lactation	0.148	0.067	2.22	***0.0292***

Results of post-hoc comparisons are presented. Cortisol concentrations are log-transformed. Results are averaged over the levels of litter size.

Table 3. Pairwise comparisons of cortisol concentrations between the domestic cat females with small and large litters at different sampling points (before mating, after parturition, 4 weeks of lactation and 8 weeks of lactation).

Sample point	Contrast	Estimate	SE	t-ratio	*p*-value
before mating	large – small	– 0.124	0.123	– 1.008	0.316
after parturition	large – small	– 0.023	0.109	– 0.214	0.831
4 weeks of lactation	large – small	– 0.198	0.109	– 1.822	0.0719
8 weeks of lactation	large – small	– 0.064	0.109	– 0.587	0.5588

Results of post-hoc comparisons are shown. Cortisol concentrations are log-transformed.

Table S4. Pairwise comparisons of cortisol concentrations at different sampling points (before mating, after parturition, 4 weeks of lactation and 8 weeks of lactation) in the domestic cat females with small and large litters.

**Contrasts**	**Females with large litters**				**Females with small litters**			
	**β**	**SE**	**t-ratio**	**p-value**	**β**	**SE**	**t-ratio**	***p*-value**
before mating –after parturition	0.012	0.099	0.125	0.9006	0.113	0.108	1.048	0.2977
before mating – 4 weeks of lactation	–0.148	0.099	–1.492	0.1394	–0.222	0.108	–2.054	0.0432
before mating – 8 weeks of lactation	–0.055	0.099	–0.555	0.5802	0.005	0.108	0.049	0.9613
after parturition – 4 weeks of lactation	–0.161	0.087	–1.843	0.069	–0.336	0.105	–3.195	0.002
after parturition – 8 weeks of lactation	–0.068	0.087	–0.776	0.4403	–0.108	0.105	–1.03	0.3063
4 weeks of lactation – 8 weeks of lactation	0.09	0.087	1.068	0.2888	0.227	0.105	2.166	0.0333

Results of post-hoc comparisons are shown. Cortisol concentrations are log-transformed. β refers to the model estimate.

Table S5. Pairwise comparisons of cortisol concentrations between the domestic cat females with different number of kittens per litter.

Contrast	Estimate	SE	t-ratio	*p*-value
1 – 2	–0.406	0.22	–1.843	0.0701
1 – 3	–0.253	0.144	–1.756	0.0824
1 – 4	–0.192	0.157	–1.223	0.2248
1 – 5	–0.064	0.131	–0.492	0.6238
1 – 6	–0.152	0.225	–0.673	0.5027
1 – 7	–0.499	0.334	–1.497	0.1407
2 – 3	0.153	0.17	0.9	0.3721
2 – 4	0.214	0.163	1.315	0.1931
2 – 5	0.342	0.169	2.021	0.0473
2 – 6	0.254	0.276	0.922	0.3601
2 – 7	–0.093	0.202	–0.46	0.6468
3 – 4	0.061	0.105	0.576	0.5663
3 – 5	0.188	0.099	1.899	0.0609
3 – 6	0.101	0.204	0.494	0.6222
3 – 7	–0.246	0.295	–0.834	0.4083
4 – 5	0.128	0.099	1.287	0.2015
4 – 6	0.041	0.202	0.2	0.8418
4 – 7	–0.307	0.273	–1.124	0.2655
5 – 6	–0.087	0.215	–0.406	0.6859
5 – 7	–0.435	0.285	–1.528	0.1321
6 – 7	–0.347	0.385	–0.902	0.3709

Results of post-hoc comparisons are presented. Cortisol concentrations are log-transformed. Results are averaged over the levels of sampling point.

Table S6. Pairwise comparisons of cortisol concentrations between the domestic cat females with different number of kittens per litter at 4 weeks of lactation.

Contrast	Estimate	SE	t-ratio	*p*-value
1 – 2	–0.201	0.254	–0.793	0.4402
1 – 3	–0.227	0.193	–1.173	0.2691
1 – 4	0.008	0.193	0.042	0.9675
1 – 5	0.224	0.182	1.232	0.2448
1 – 6	–0.222	0.334	–0.665	0.5187
1 – 7	–0.07	0.37	–0.188	0.8535
2 – 3	–0.025	0.192	–0.131	0.8973
2 – 4	0.21	0.209	1.001	0.3325
2 – 5	0.426	0.209	2.034	0.0584
2 – 6	–0.021	0.345	–0.06	0.9526
2 – 7	0.132	0.273	0.483	0.6378
3 – 4	0.234	0.13	1.806	0.0981
3 – 5	0.451	0.133	3.398	0.0061
3 – 6	0.004	0.296	0.015	0.9886
3 – 7	0.157	0.336	0.468	0.6472
4 – 5	0.216	0.129	1.671	0.1227
4 – 6	–0.23	0.276	–0.834	0.4205
4 – 7	–0.078	0.338	–0.23	0.8213
5 – 6	–0.447	0.31	–1.439	0.1739
5 – 7	–0.294	0.333	–0.881	0.392
6 – 7	0.153	0.471	0.325	0.7511

Results of post-hoc comparisons are presented. Cortisol concentrations are log-transformed.

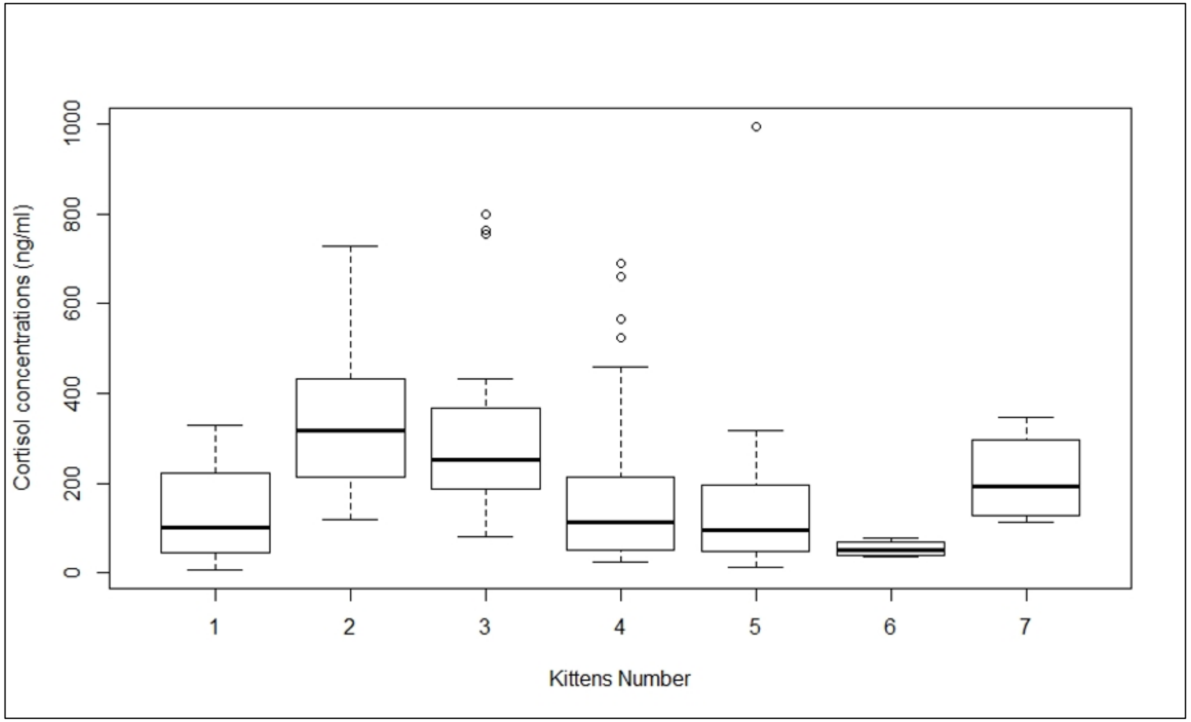

Figure S3. Cortisol concentrations in females of the domestic cat with different number of kittens per litter. Plotted are the median (horizontal line in the box), lower and upper quartiles (horizontal box boundaries), minimum and maximum values (whiskers).
